# Clinician Acceptance of Artificial Intelligence- and Extended Reality-Enabled Telemedicine: A Cross-Sectional Vignette Survey of Residents and Nurses in Romania

**DOI:** 10.3390/jcm15103565

**Published:** 2026-05-07

**Authors:** Codrina Mihaela Levai, Livia Stanga, Laura Alexandra Nussbaum, Adelina Marioara Gherman, Daian-Ionel Popa, Camelia Fizedean

**Affiliations:** 1 Doctoral School, “Victor Babes” University of Medicine and Pharmacy, 300041 Timisoara, Romania; codrinalevai@umft.ro (C.M.L.); daian-ionel.popa@umft.ro (D.-I.P.); 2 Research Center for Medical Communication, “Victor Babes” University of Medicine and Pharmacy, 300041 Timisoara, Romania; 3 Discipline of Microbiology, Faculty of Medicine, “Victor Babes” University of Medicine and Pharmacy, 300041 Timisoara, Romania; 4 Department of Neurosciences, “Victor Babes” University of Medicine and Pharmacy, 300041 Timisoara, Romania; fizedean.camelia@umft.ro; 5 Faculty of Nursing, “Victor Babes” University of Medicine and Pharmacy, 300041 Timisoara, Romania; 6 Multidisciplinary Heart Research Center, “Victor Babes” University of Medicine and Pharmacy, 300041 Timisoara, Romania

**Keywords:** telemedicine, artificial intelligence, extended reality, clinician acceptance, technostress, health literacy, vignette survey

## Abstract

**Background and Objectives**: Artificial intelligence (AI) and extended reality (XR) are increasingly being integrated into telemedicine, yet clinician adoption depends not only on perceived utility but also on digital preparedness and technology-related burden. This study compared clinician acceptance of AI-only, XR-only, and combined AI+XR telemedicine scenarios and examined whether AI literacy, health literacy, technostress, age, and sex explained variability in acceptance. **Materials and Methods**: We conducted a cross-sectional, anonymous online vignette survey among 117 resident physicians and nurses from a tertiary-care hospital and affiliated outpatient clinics in Western Romania. Participants evaluated three randomized telemedicine scenarios (AI-only, XR-only, and AI+XR) using a 3-item Acceptance Index scored from 1 to 4. Additional measures included a study-developed AI literacy quiz, the Romanian-validated HLS-EU-Q16, an adapted brief technostress scale, prior AI/XR exposure, perceived accessibility, perceived value, privacy concern, and demographic variables. **Results**: Acceptance was highest for the combined AI+XR scenario (3.74 ± 0.49), followed by AI-only (3.64 ± 0.56) and XR-only (3.49 ± 0.64). AI+XR acceptance was significantly higher than AI-only and XR-only acceptance (both *p* < 0.001), although the absolute between-scenario differences were modest. Residents reported consistently higher acceptance than nurses across all scenarios, whereas sex differences were small and non-significant; younger age showed only weak inverse associations with acceptance. AI+XR acceptance correlated positively with AI literacy (ρ = 0.60) and health literacy (ρ = 0.23), and negatively with technostress (ρ = −0.47). In multivariable analysis, higher AI literacy (β = 0.603, *p* < 0.001) and health literacy (β = 0.241, *p* < 0.001) independently predicted higher AI+XR acceptance, whereas technostress was inversely associated (β = −0.212, *p* < 0.001). **Conclusions**: In this sample, clinicians rated the integrated AI+XR vignette most favorably, but the observed differences between scenarios should be interpreted cautiously and as hypothesis-generating rather than definitive evidence of superiority. Acceptance appeared to depend more on digital readiness and technostress than on age or sex, supporting implementation strategies focused on literacy-building, workflow fit, and burden reduction.

## 1. Introduction

Digital health has shifted from a supportive add-on to a core delivery channel for outpatient care, with telemedicine scaling rapidly as health systems seek to improve access, continuity, and resilience during public health disruptions. Global guidance emphasizes that sustained digital transformation requires not only technology procurement but also governance, workforce readiness, and implementation capacity across clinical settings [1,2].

Within telemedicine, artificial intelligence (AI) is increasingly embedded in triage, risk stratification, decision support, and documentation workflows, promising to augment clinician performance and reduce cognitive burden. At the same time, the clinical impact of AI depends on reliability, transparency, and safe integration into real-world care pathways rather than stand-alone model accuracy [3,4].

Extended reality (XR), including augmented reality (AR) and virtual reality (VR), has complementary potential for remote examination, procedural guidance, and clinician–patient interaction through immersive or overlay interfaces. However, telemedicine innovations frequently encounter nonadoption or abandonment when complexity, workflow misfit, and organizational constraints outweigh perceived benefits, underscoring the need for pragmatic evaluation frameworks [5].

In the European Union, clinician-facing AI/XR telemedicine also operates within a dense compliance environment. The General Data Protection Regulation (GDPR) governs processing of health data, the Medical Device Regulation (MDR) applies to software qualifying as a medical device, and the EU Artificial Intelligence Act establishes risk-based requirements for high-risk AI systems relevant to healthcare [6,7,8]. In parallel, ethical guidance on trustworthy AI and international recommendations highlight privacy, transparency, safety, accountability, and human oversight as prerequisites for clinical trust [9,10,11,12,13,14,15].

Adoption is additionally shaped by perceived usefulness, ease of use, and facilitating conditions, as described by established acceptance models [16,17,18,19,20,21]. For clinicians, technology-related overload, complexity, and intrusion (technostress) may directly suppress willingness to use telemedicine tools even when their clinical value is recognized [22,23,24,25]. Using STROBE-aligned reporting for observational research and a validated Romanian health literacy instrument, this cross-sectional study aims to (i) quantify acceptance of AI-only, XR-only, and combined AI+XR telemedicine scenarios among residents and nurses; and (ii) test whether AI literacy, health literacy, and technostress jointly explain heterogeneity in acceptance.

## 2. Materials and Methods

### 2.1. Study Design & Setting

Cross-sectional, anonymous, vignette-based online survey administered in Romania between 10 March and 15 May 2025. Recruitment was conducted at a tertiary-care hospital and affiliated outpatient clinics in Western Romania. Eligible clinicians worked in services in which telemedicine workflows were already used for remote follow-up or were institutionally eligible for near-term deployment; routine day-to-day use of AI/XR tools was not required for inclusion. To distinguish workplace eligibility from previous hands-on exposure, the questionnaire separately captured prior use of AI tools and prior use of XR technologies, and these variables were explored descriptively and in multivariable analysis. Participation was voluntary and based on electronic informed consent. No identifiable personal data were collected. The protocol was approved by the institutional ethics committee of the Victor Babes University of Medicine and Pharmacy Timisoara prior to data collection, and all procedures were designed to comply with applicable data-protection requirements.

### 2.2. Participants and Sample Size

Eligible participants were clinicians aged ≥ 18 years who were either resident physicians or registered nurses at the participating institutions and who could complete a Romanian-language survey. Participants were excluded if they did not complete the core acceptance items for at least one vignette or if the response pattern suggested invalid completion (e.g., straight-lining across all acceptance items together with implausibly short completion time).

A total of 146 invitation emails were distributed through departmental mailing lists (71 to residents and 75 to nurses). Of these, 120 questionnaires were returned; three were excluded for missing core acceptance items, leaving 117 complete responses for analysis (overall response rate, 80.1%; completion rate among submitted questionnaires, 97.5%). Recruitment used convenience sampling, aiming for an approximately balanced distribution between residents and nurses. Because the survey was anonymous and invitations were distributed without person-level tracking, demographic characteristics of non-responders could not be collected.

### 2.3. Measures and Survey Instrument

The survey included three short telemedicine vignettes presented in randomized order on separate screens. Each vignette contained the same clinical frame—a remote outpatient interaction involving assessment, triage, and follow-up—but varied the technological component being described: (i) AI-only decision support embedded in a video visit, (ii) XR-only support through AR/VR-assisted remote examination or procedural visualization, and (iii) a combined AI+XR scenario integrating decision support with immersive or overlay-based interaction. After reading each scenario, participants immediately completed the acceptance items before moving to the next screen, which reduced carry-over between responses. To improve scenario transparency, Table 1 summarizes the clinical frame and specifies what XR and AI contributed in each vignette, while preserving the same baseline remote outpatient context across conditions.

For each vignette, acceptance was measured using a study-developed 3-item Acceptance Index scored on a 1–4 Likert scale (trust in the tool, perceived improvement in care, and willingness to use). The items were informed by technology-acceptance constructs related to usefulness, trust, and behavioral intention [11,12] and were reviewed for content relevance by two clinical academics and one digital-health researcher before pilot testing. Scenario-level scores were computed as the mean of the three items, with higher scores indicating greater acceptance.

Determinants included: (i) an 8-item study-developed AI literacy quiz (0–8), designed to capture basic conceptual knowledge relevant to telemedicine applications; (ii) the Romanian-validated HLS-EU-Q16 health literacy instrument (0–16) [15]; and (iii) a 4-item brief technostress scale adapted from prior technostress literature for the clinical digital-workflow context [13,25]. The study-developed sections were piloted in 12 clinicians (6 residents and 6 nurses) not included in the final analysis to refine wording, clarity, and face validity. Additional covariates included prior use of AI tools and XR technologies (captured separately as previous hands-on exposure), perceived accessibility and perceived value of telemedicine (1–4), privacy concern (1–4), and basic demographics (age, sex, professional role).

### 2.4. Data Collection, Outcomes, and Data Management

The survey was deployed on a secure web-based platform between 10 March and 15 May 2025 and required approximately 8–12 min to complete. Responses were stored without IP addresses or device identifiers. To minimize missingness, key items were set as required; nonetheless, participants could exit at any time without penalty.

The primary outcome was acceptance of the AI+XR vignette, operationalized as the scenario-level Acceptance Index. Secondary outcomes included acceptance of AI-only and XR-only vignettes, within-participant differences between scenarios, and exploratory subgroup differences by age, sex, and professional role. Data were exported to a locked analysis environment and underwent range checks, duplicate screening, and review of completion-time outliers before analysis.

### 2.5. Statistical Analysis

Internal consistency of each scenario’s Acceptance Index and of the adapted technostress scale was assessed using Cronbach’s α; the knowledge-based AI literacy quiz was summarized as a total correct score. Analyses were conducted in R version 4.3.2 (R Foundation for Statistical Computing, Vienna, Austria). Scenario effects were evaluated using within-participant paired *t*-tests. For these comparisons, we additionally report mean differences with 95% confidence intervals and Cohen’s dz effect sizes. Associations between acceptance and candidate determinants were explored using Spearman correlations. Differences by sex were examined with independent-samples tests, and age was analyzed both continuously (Spearman coefficients) and descriptively by age category (<30 vs. ≥30 years) overall and within residents and nurses. The primary multivariable model predicted AI+XR acceptance using robust (HC3) standard errors and included AI literacy, health literacy, their interaction term (AI literacy × health literacy), technostress, perceived accessibility, perceived value, privacy concern, prior AI use, prior XR use, age, professional role, and sex. To characterize heterogeneity, exploratory k-means clustering (k = 3) was applied to standardized acceptance, literacy, and technostress features, and cluster profiles were summarized descriptively. Statistical significance was interpreted at a two-sided α = 0.05 without formal adjustment for multiplicity for the exploratory analyses.

## 3. Results

Of 146 invitation emails distributed through departmental mailing lists (71 to residents and 75 to nurses), 120 questionnaires were returned and 117 complete responses were retained for analysis, yielding an overall response rate of 80.1% and a completion rate of 97.5% among submitted questionnaires. Because participation was anonymous and invitations were not linked to individual identifiers, detailed characteristics of non-responders were not available. Internal consistency of the 3-item Acceptance Index was high across the three scenarios (Cronbach’s α: AI-only = 0.893; XR-only = 0.896; AI+XR = 0.892), and the adapted technostress scale also showed acceptable reliability (α = 0.81). Table 2 presents the sample profile. Participants had a mean age of 30.6 ± 5.2 years and were predominantly female (79/117; 67.5%). Residents represented 50.4% (59/117) and were younger than nurses (28.4 ± 2.7 vs. 32.8 ± 5.9 years). Prior exposure to AI tools was reported by 43.6% (51/117), whereas prior XR use was less common (13.7%; 16/117). Mean AI literacy was 5.03 ± 1.79 (0–8), health literacy (HLS-EU-Q16) was 13.68 ± 1.77 (0–16), and technostress averaged 3.03 ± 0.72 (1–5).

Table 3 summarizes scenario-level acceptance (1–4 scale) across demographic and professional subgroups. Acceptance was highest for the combined AI+XR vignette (total 3.74 ± 0.49), followed by AI-only (3.64 ± 0.56) and XR-only (3.49 ± 0.64). Residents consistently reported higher acceptance than nurses across scenarios (e.g., AI+XR: 3.87 ± 0.37 vs. 3.61 ± 0.56). Descriptively, participants aged < 30 years and male participants reported slightly higher acceptance scores than their counterparts, but these differences were small. In formal analyses, age showed weak inverse correlations with AI-only (ρ = −0.18, *p* = 0.049) and AI+XR acceptance (ρ = −0.21, *p* = 0.023), but not XR-only acceptance (ρ = −0.14, *p* = 0.127). Within residents, age was not associated with acceptance for AI-only, XR-only, or AI+XR (all *p* > 0.35), whereas within nurses a modest inverse association emerged for AI+XR (ρ = −0.27, *p* = 0.041). Sex differences were not statistically significant overall (AI-only *p* = 0.312; XR-only *p* = 0.289; AI+XR *p* = 0.274) or when residents and nurses were analyzed separately (all *p* > 0.40). Prior AI use was associated with slightly higher unadjusted AI+XR acceptance (3.82 ± 0.41 vs. 3.68 ± 0.54), whereas prior XR use showed minimal difference (3.77 ± 0.46 vs. 3.73 ± 0.50); neither exposure variable altered the overall preference ordering, and neither remained independently significant in multivariable analysis.

Table 4 shows within-participant paired comparisons between scenarios together with 95% confidence intervals and effect sizes. Acceptance for AI+XR exceeded AI-only by 0.10 ± 0.27 (95% CI 0.05 to 0.15; t = 4.03; *p* < 0.001; dz = 0.37) and exceeded XR-only by 0.25 ± 0.35 (95% CI 0.19 to 0.32; t = 7.58; *p* < 0.001; dz = 0.70). AI-only also outperformed XR-only (Δ = 0.15 ± 0.30; 95% CI 0.09 to 0.20; t = 5.26; *p* < 0.001; dz = 0.49), indicating a statistically significant but mostly small-to-moderate graded preference for integrated AI+XR support.

Table 5 reports Spearman correlations among key variables. AI+XR acceptance correlated positively with AI literacy (ρ = 0.60) and perceived value (ρ = 0.26), and negatively with technostress (ρ = −0.47). Health literacy showed a modest positive association with acceptance (ρ = 0.23), while privacy concern had a near-zero association (ρ = −0.04). Given the deliberately concise vignette format, this latter finding should be interpreted cautiously and not as evidence that privacy considerations are unimportant in real clinical implementation.

Table 6 presents the multivariable regression for AI+XR acceptance using robust (HC3) standard errors. Higher AI literacy (standardized β = 0.603; 95% CI 0.347 to 0.859; *p* < 0.001) and higher health literacy (β = 0.241; 95% CI 0.127 to 0.355; *p* < 0.001) were independently associated with higher acceptance, whereas technostress was inversely associated (β = −0.212; 95% CI −0.323 to −0.101; *p* < 0.001). The AI literacy × health literacy interaction term was negative (β = −0.035; 95% CI −0.055 to −0.016; *p* < 0.001), indicating that the marginal association of AI literacy with acceptance varied by health literacy level. Age, sex, professional role, prior AI use, and prior XR use were not statistically significant after adjustment (all *p* ≥ 0.198). The model explained a substantial proportion of variance in AI+XR acceptance (adjusted R^2^ = 0.49).

Table 7 summarizes exploratory clustering (k = 3) based on acceptance and key determinants. Cluster 1 showed lower acceptance (mean 2.82 ± 0.39) with low AI literacy (2.95 ± 1.17) and higher technostress (3.55 ± 0.63). Clusters 2 and 3 both had very high acceptance (3.93 ± 0.15 and 3.99 ± 0.05), but differed in profiles: Cluster 2 combined high health literacy (15.09 ± 0.92) with moderate technostress (3.20 ± 0.60), whereas Cluster 3 had the highest AI literacy (5.80 ± 1.45) and the lowest technostress (2.53 ± 0.61).

**Figure 1:**
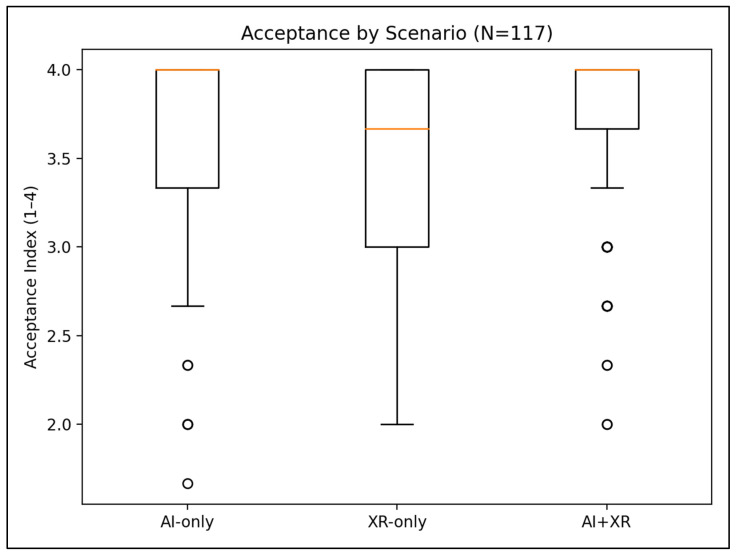
Acceptance by scenario (boxplots).

Figure 1 visualizes acceptance distributions across scenarios. The boxplots show a right-shift in scores for the combined AI+XR scenario relative to AI-only and XR-only, consistent with the mean differences in Table 4; dispersion is also narrower for AI+XR, suggesting more consistently favorable evaluations across participants.

**Figure 2:**
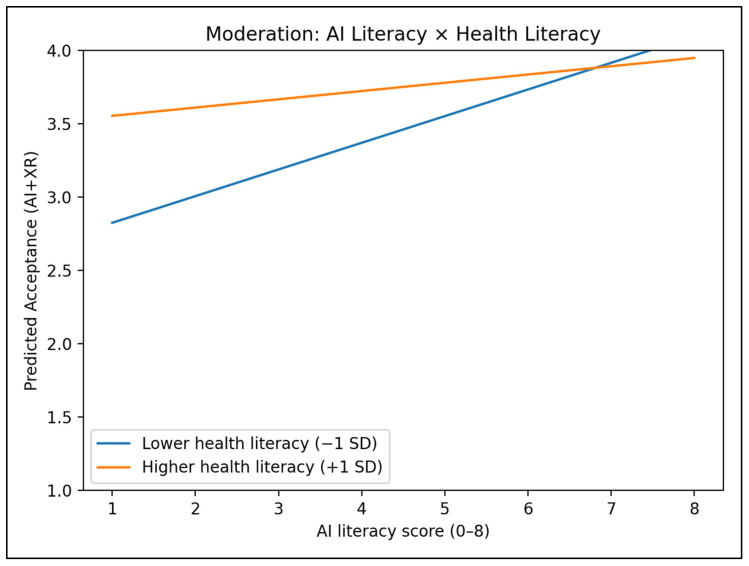
Moderation plot: predicted AI+XR acceptance by AI literacy at low vs. high health literacy.

Figure 2 illustrates the moderation pattern for AI+XR acceptance. Predicted acceptance increases with AI literacy across the observed range, with separation between low and high health literacy conditions; the non-parallel trend is consistent with the significant interaction term in Table 6.

## 4. Discussion

### 4.1. Analysis of Findings

Across the three modality scenarios, participants expressed the strongest preference for integrated AI+XR support, with AI-only scoring slightly higher than XR-only. This pattern suggests that clinicians may respond more favorably when decision support and interface enhancement are presented as part of the same workflow rather than as disconnected digital layers [16,17,18]. At the same time, the absolute difference between AI+XR and AI-only was only 0.10 points on a 4-point scale, with a small effect size (dz = 0.37). For that reason, the present data support a relative preference, not a large or clinically decisive superiority of combined platforms.

The multivariable findings point to two practical levels: capability and burden. Higher AI literacy and higher health literacy were independently associated with greater acceptance, whereas technostress was inversely associated with acceptance. This pattern is in line with prior work showing that clinicians are more receptive to AI when they understand what the tool does, how to interpret its output, and how it fits into clinical reasoning, but become more hesitant when digital systems add complexity, interruption, or workflow friction [21,22,23,24,25].

The interaction between AI literacy and health literacy suggests that technical familiarity alone is not enough. Adoption likely depends on whether clinicians can integrate digital information into patient communication, decision-making, and professional judgment. This interpretation is consistent with behavioral research showing that even accurate or potentially useful medical AI may be resisted when it is perceived as opaque, autonomy-threatening, or misaligned with human oversight [19,20,24].

Age and sex contributed relatively little to acceptance in this cohort. Younger age showed only weak inverse associations overall and mainly within the nursing subgroup, while sex differences were consistently small and non-significant. Because the sample was relatively young and heavily composed of trainees, these subgroup findings should be interpreted cautiously; they do not rule out stronger age-related gradients in older or more senior clinical populations [24,25,26,27].

The minimal association between privacy concern and acceptance should also be read cautiously. Our vignettes were intentionally brief and focused on workflow scenarios rather than detailed data-sharing architectures, consent pathways, or cybersecurity safeguards. Accordingly, the near-zero correlation probably reflects measurement simplification rather than proof that privacy is irrelevant. In real implementation settings, trust, governance transparency, and data stewardship are likely to remain central determinants of adoption [26,28].

These results therefore should be viewed as hypothesis-generating and implementation-oriented. They suggest that integrated AI+XR telemedicine may merit pragmatic evaluation in settings where both decision support and enhanced interaction are clinically meaningful, but they do not justify a blanket recommendation to prioritize combined platforms in all contexts. The most plausible practical implication is that acceptance can be improved by pairing technology deployment with literacy-building, low-burden interface design, profession-specific onboarding, and clearly articulated human oversight. Specialty-specific success stories, such as AI-assisted ECG interpretation, arrhythmia detection, electroanatomical mapping, and ablation planning in cardiac electrophysiology, further illustrate how clinician acceptance is likely to increase when AI is tied to concrete, high-value tasks rather than abstract digital novelty [29,30,31].

### 4.2. Study Limitations

Several limitations should be considered when interpreting these findings. First, the cross-sectional design precludes causal inference, so the observed associations between AI literacy, health literacy, technostress, and acceptance cannot establish directionality. Second, the study relied on a convenience sample of 117 clinicians from a tertiary hospital and affiliated clinics in Western Romania. The sample was relatively young and included many trainees, which may have increased baseline openness to digital tools and limits generalizability to older, more experienced, or differently resourced clinical workforces. Third, although the response rate was acceptable, characteristics of non-responders could not be analyzed because the anonymous distribution procedure did not allow person-level linkage. Fourth, acceptance was measured using vignette-based scenarios rather than observation of real-world use of deployed AI/XR telemedicine tools; therefore, the results may reflect stated intention more than actual behavior, and an intention–behavior gap remains possible. Fifth, several measures were study-developed or adapted for this context, so some degree of measurement error or construct simplification cannot be excluded despite pilot testing and acceptable internal consistency. Finally, the exploratory clustering results should be interpreted cautiously because cluster solutions may be sample-dependent and require external validation in larger, multisite studies that include more detailed organizational, specialty-specific, and infrastructural variables.

## 5. Conclusions

Acceptance of telemedicine was highest for the combined AI+XR scenario, intermediate for the AI-only scenario, and lowest for the XR-only scenario, indicating that clinicians in this sample expressed the most favorable views toward digitally integrated solutions. However, the absolute differences between scenarios were modest and should not be interpreted as definitive evidence that combined platforms are clinically superior. Acceptance of AI+XR was positively associated with AI literacy and health literacy and inversely associated with technostress, while age and sex contributed little to explained variance after adjustment. Together, these findings suggest that clinician adoption of emerging telemedicine modalities is shaped more by digital readiness and workflow burden than by basic demographic characteristics. The study therefore offers hypothesis-generating evidence that implementation efforts may benefit from literacy-building, technostress mitigation, and profession-sensitive deployment strategies. Future studies should test these mechanisms in larger and more diverse clinical settings and link acceptance measures to actual utilization and patient-care outcomes.

## Figures and Tables

**Table 1:** Scenario table clarifying the clinical frame and technological contribution of each telemedicine vignette.

Scenario Name/Clinical Context	XR Component	AI Component
AI-only decision-supported video visit: a remote outpatient assessment, triage, and follow-up encounter in which the clinician evaluates a patient with a new or worsening ambulatory complaint and decides whether routine follow-up, expedited review, or in-person assessment is needed.	Not included; the interaction remains a standard video visit without immersive or overlay-based examination support.	Embedded AI decision support summarizes patient-reported symptoms, flags severity or risk features, suggests differential diagnostic considerations, and proposes follow-up urgency and management options for clinician review.
XR-only AR/VR-assisted remote examination: the same remote outpatient encounter, with emphasis on visualizing examination steps and guiding patient or caregiver positioning during a telemedicine assessment.	AR/VR support provides overlay-based guidance for the remote examination, visualization of relevant anatomy or procedural steps, and structured prompts to support examination completeness.	Not included; triage, diagnosis, and follow-up decisions remain based on clinician judgment without algorithmic recommendations.
Integrated AI+XR telemedicine encounter: the same outpatient assessment, triage, and follow-up frame using both decision support and immersive or overlay-based interaction during the visit.	AR/VR support guides examination maneuvers, helps visualize clinical findings or procedural steps, and enables a more structured remote interaction.	AI decision support integrates the history and vignette findings, provides risk stratification and diagnostic or management suggestions, and recommends follow-up priority, while the clinician retains final responsibility.

**Table 2:** Sample characteristics stratified by professional role.

	Total (N = 117)	Residents (*n* = 59)	Nurses (*n* = 58)
Age (years), mean ± SD	30.6 ± 5.2	28.4 ± 2.7	32.8 ± 5.9
Female, ***n*** (%)	79 (67.5%)	37 (62.7%)	42 (72.4%)
Male, ***n*** (%)	38 (32.5%)	22 (37.3%)	16 (27.6%)
Prior AI use, ***n*** (%)	51 (43.6%)	31 (52.5%)	20 (34.5%)
Prior XR use, ***n*** (%)	16 (13.7%)	11 (18.6%)	5 (8.6%)
AI literacy (0–8), mean ± SD	5.03 ± 1.79	5.64 ± 1.48	4.41 ± 1.89
Health literacy HLS-EU-Q16 (0–16), mean ± SD	13.68 ± 1.77	13.92 ± 1.64	13.43 ± 1.87
Technostress (1–5), mean ± SD	3.03 ± 0.72	2.86 ± 0.68	3.20 ± 0.72

**Table 3:** Acceptance Index (1–4) across modality scenarios and demographic/professional subgroups.

Group	AI-Only	XR-Only	AI+XR
Total (N = 117)	3.64 ± 0.56	3.49 ± 0.64	3.74 ± 0.49
Female (*n* = 79)	3.61 ± 0.58	3.45 ± 0.66	3.71 ± 0.51
Male (*n* = 38)	3.70 ± 0.51	3.57 ± 0.59	3.80 ± 0.43
Age < 30 years (*n* = 49)	3.71 ± 0.49	3.56 ± 0.58	3.81 ± 0.44
Age ≥ 30 years (*n* = 68)	3.58 ± 0.60	3.44 ± 0.68	3.69 ± 0.52
Residents (*n* = 59)	3.81 ± 0.47	3.67 ± 0.54	3.87 ± 0.37
Nurses (*n* = 58)	3.47 ± 0.59	3.31 ± 0.69	3.61 ± 0.56

**Table 4:** Paired comparisons between scenarios (Acceptance Index) with effect sizes.

	Mean Δ ± SD	95% CI	t	*p*	Cohen’s dz
AI+XR − AI-only	0.10 ± 0.27	0.05 to 0.15	4.03	<0.001	0.37
AI+XR − XR-only	0.25 ± 0.35	0.19 to 0.32	7.58	<0.001	0.70
AI-only − XR-only	0.15 ± 0.30	0.09 to 0.20	5.26	<0.001	0.49

**Table 5:** Spearman correlations among key variables.

	Acceptance_AI+XR	AI_Literacy_0_8	HLS_EU_Q16_0_16	Technostress_1_5	Accessibility_1_4	Perceived_Value_1_4	Privacy_Concern_1_4
Acceptance_AI+XR	1.0	0.6	0.23	−0.47	0.18	0.26	−0.04
AI_Literacy_0_8	0.6	1.0	0.05	−0.16	0.0	0.18	−0.01
HLS_EU_Q16_0_16	0.23	0.05	1.0	0.04	−0.02	0.07	−0.01
Technostress_1_5	−0.47	−0.16	0.04	1.0	−0.25	−0.16	0.03
Accessibility_1_4	0.18	0.0	−0.02	−0.25	1.0	0.03	0.07
Perceived_Value_1_4	0.26	0.18	0.07	−0.16	0.03	1.0	0.08
Privacy_Concern_1_4	−0.04	−0.01	−0.01	0.03	0.07	0.08	1.0

**Table 6:** Multivariable regression predicting AI+XR acceptance (HC3 SE, standardized coefficients).

Predictor	Standardized β (HC3 SE)	95% CI	*p*
AI_Literacy_0_8	0.603 (0.130)	0.347 to 0.859	<0.001
HLS_EU_Q16_0_16	0.241 (0.058)	0.127 to 0.355	<0.001
AIxHLS	−0.035 (0.010)	−0.055 to −0.016	<0.001
Technostress_1_5	−0.212 (0.056)	−0.323 to −0.101	<0.001
Accessibility_1_4	0.029 (0.064)	−0.097 to 0.155	0.658
Perceived_Value_1_4	0.076 (0.060)	−0.043 to 0.195	0.200
Privacy_Concern_1_4	0.012 (0.052)	−0.091 to 0.115	0.821
Prior_AI	0.106 (0.079)	−0.051 to 0.263	0.178
Prior_XR	0.041 (0.091)	−0.139 to 0.221	0.651
Age	−0.009 (0.007)	−0.023 to 0.005	0.198
Resident	0.028 (0.080)	−0.129 to 0.185	0.729
Female	−0.054 (0.065)	−0.182 to 0.074	0.404

**Table 7:** Cluster profiles (k = 3) based on acceptance, AI literacy, health literacy, and technostress.

	Acceptance_AI+XR Mean	Acceptance_AI+XR std	AI_Literacy_0_8 Mean	AI_Literacy_0_8 std	HLS_EU_Q16_0_16 Mean	HLS_EU_Q16_0_16 std	Technostress_1_5 Mean	Technostress_1_5 std
1	2.82	0.39	2.95	1.17	12.45	1.63	3.55	0.63
2	3.93	0.15	5.28	1.6	15.09	0.92	3.2	0.6
3	3.99	0.05	5.8	1.45	12.46	1.25	2.53	0.61

## Data Availability

The data presented in this study are available on request from the corresponding author.
